# Detection of Acute Brain Injury in Intensive Care Unit Patients on ECMO Support Using Ultra-Low-Field Portable MRI: A Retrospective Analysis Compared to Head CT

**DOI:** 10.3390/diagnostics14060606

**Published:** 2024-03-13

**Authors:** Sung-Min Cho, Shivalika Khanduja, Jiah Kim, Jin Kook Kang, Jessica Briscoe, Lori R. Arlinghaus, Kha Dinh, Bo Soo Kim, Haris I. Sair, Audrey-Carelle N. Wandji, Elena Moreno, Glenda Torres, Jose Gavito-Higuera, Huimahn A. Choi, John Pitts, Aaron M. Gusdon, Glenn J. Whitman

**Affiliations:** 1 Division of Cardiac Surgery, Department of Surgery, Johns Hopkins University School of Medicine, Baltimore, MD 21287, USA; 2 Department of Neurology, Johns Hopkins University School of Medicine, Baltimore, MD 21205, USA; 3 Division of Neuroscience Critical Care, Departments of Neurosurgery, Anesthesiology, Critical Care Medicine, The Johns Hopkins Hospital, Baltimore, MD 21287, USA; 4 Hyperfine, Inc., Guilford, CT 06437, USA; 5 Divisions of Pulmonary, Critical Care and Sleep Medicine, The University of Texas Health Science Center at Houston, Houston, TX 77030, USA; 6 Department of Advanced Cardiopulmonary Therapies and Transplantation, The University of Texas Health Science Center at Houston, Houston, TX 77030, USA; 7 Division of Pulmonary and Critical Care Medicine, Johns Hopkins Medicine, Baltimore, MD 21205, USA; 8 Division of Neuroradiology, The Russell H. Morgan Department of Radiology and Radiological Science, Johns Hopkins University School of Medicine, Baltimore, MD 21287, USA; 9 The Malone Center for Engineering in Healthcare, The Whiting School of Engineering, Johns Hopkins University, Baltimore, MD 21218, USA; 10 Division of Neurocritical Care, Department of Neurosurgery, McGovern School of Medicine, The University of Texas Health Science Center at Houston, Houston, TX 77030, USA; 11 Department of Diagnostic and Interventional Imaging, McGovern Medical School, The University of Texas Health Science Center at Houston, Houston, TX 77030, USA

**Keywords:** acute brain injury, critical care, extracorporeal membrane oxygenation, neuroimaging, portable MR

## Abstract

Early detection of acute brain injury (ABI) is critical to intensive care unit (ICU) patient management and intervention to decrease major complications. Head CT (HCT) is the standard of care for the assessment of ABI in ICU patients; however, it has limited sensitivity compared to MRI. We retrospectively compared the ability of ultra-low-field portable MR (ULF-pMR) and head HCT, acquired within 24 h of each other, to detect ABI in ICU patients supported on extracorporeal membrane oxygenation (ECMO). A total of 17 adult patients (median age 55 years; 47% male) were included in the analysis. Of the 17 patients assessed, ABI was not observed on either ULF-pMR or HCT in eight patients (47%). ABI was observed in the remaining nine patients with a total of 10 events (8 ischemic, 2 hemorrhagic). Of the eight ischemic events, ULF-pMR observed all eight, while HCT only observed four events. Regarding hemorrhagic stroke, ULF-pMR observed only one of them, while HCT observed both. ULF-pMR outperformed HCT for the detection of ABI, especially ischemic injury, and may offer diagnostic advantages for ICU patients. The lack of sensitivity to hemorrhage may improve with modification of the imaging acquisition program.

## 1. Introduction

The accurate detection of cerebral complications, notably acute brain injury (ABI), in critically ill patients is vital to the timely administration of brain-saving interventions. Sophisticated equipment for the hemodynamic monitoring of patients in the intensive care unit (ICU) has allowed for the early detection of complications [1]. Additional observation of neurological status has been shown to improve overall outcomes in ICU patients [2]; however, obtaining advanced neuroimaging in ICU patients is often challenging. In particular, magnetic resonance imaging (MRI) provides added value over head-computed tomography (HCT) for lesion detection in a variety of pathologies [3]. However, traditional MRI systems have large fringe fields that prohibit the concomitant use of essential monitoring and life-saving devices, such as extracorporeal membrane oxygenation (ECMO). In addition, transporting these patients outside of the ICU to radiology to undergo standard-of-care HCT can lead to adverse events (i.e., hypoxia and hypotension) due to uncontrolled conditions and position changes during transport [4,5,6,7].

The recent introduction of an FDA-cleared portable, ultra-low field (0.064 Tesla) MR (ULF-pMR) system (*Swoop*^®^, Hyperfine, Inc., Guilford, CT, USA) has enabled soft-tissue contrast imaging with MRI at the ICU patient’s bedside [8,9,10,11,12,13,14,15,16,17,18]. Previous evidence suggests that ULF-pMR can offer additional information [2] and serial imaging for ICU patients to aid in acute diagnoses and management [15]. However, an assessment of ULF-pMR compared to standard-of-care HCT to detect ABI in ICU patients has not been reported.

Herein, we aimed to compare the ability to detect ABI in ICU patients on ECMO who underwent both ULF-pMR and HCT within a 24 h window as recommended by an ongoing prospective observational study (SAFE MRI study: NCT05469139). We hypothesized that ULF-pMR would outperform HCT for the diagnosis of ABI, especially for ischemic events.

## 2. Materials and Methods

The study was conducted following the Declaration of Helsinki and approved by the Institutional Review Boards of The Johns Hopkins Medicine (IRB00285716 approved on 8 October 2021) and the University of Texas-Houston (HSC-MS-22-0608 approved on 11 July 2022). Informed consent was obtained from a legally authorized representative as enrolled patients were unable to provide consent.

Participants were adults enrolled as part of an ongoing multicenter prospective observational study (SAFE MRI study: NCT05469139). Inclusion criteria included adult patients (≥18 years) with venoarterial (VA) or venovenous (VV) ECMO. Exclusion criteria included specific contraindications for MRI, patient weight exceeding 440 lbs (200 kg), implanted ventricular assist device (VAD) or Percutaneous Ventricular Assist Device, and pregnancy. This retrospective study included a subset of patients who underwent both ULF-pMR and HCT within a 24 h window.

The detailed study procedure was described in our original protocol [12]; however, pertinent details are listed here. The ULF-pMR (*Swoop*^®^ 1.6 and 1.8, Hyperfine, Inc., Guilford, CT, USA) was positioned at the head of the patient’s bed. All ICU equipment was positioned to be outside of the 5 Gauss line. Trained staff moved the patient to position the patient’s head inside the head coil using a lift-and-slide maneuver. Vital signs, ECMO flow, and positioning of the cannula and the endotracheal tube were monitored continuously.

T1-weighted, T2-weighted, fluid-attenuated inversion recovery (FLAIR), and diffusion-weighted imaging with automatically calculated apparent diffusion coefficient map sequences were acquired (software versions 8.6.1 and rc8.7, Hyperfine, Inc., Guilford, CT, USA). HCT was acquired as part of a standardized neuromonitoring protocol [2] using either a Somatom (Siemens Healthineers AG, Forchheim, Germany) or Aquilion ONE GENESIS Edition (Canon Medical Systems USA, Inc., Tustin, CA, USA) CT scanner. Both exams were reviewed by a board-certified neuroradiologist, and the presence of ABI was recorded.

We compared the ABI (ischemic and hemorrhagic events) detection/diagnosis between ULF-pMRI and HCT, summarizing the results with descriptive statistics. In addition, the sensitivity, specificity, positive predictive value, and negative predictive value of ULF-pMR for ischemic and hemorrhagic events were calculated, using HCT as the reference standard since conventional MR was not available. Agreement between ULF-pMR and HCT for ischemic and hemorrhagic events was assessed by calculating Cohen’s kappa, and the results were interpreted as follows [19]: 0–0.2: none; 0.21–0.39: minimal; 0.4–0.59: weak; 0.6–0.79: moderate; 0.8–0.9: strong; >0.9: almost perfect.

## 3. Results

Out of an initial cohort of 46 patients, 35 (76%) were scanned using both ULF-pMR and HCT during their ECMO support. The final cohort included in this assessment consisted of 17 patients (37%) who underwent both diagnostic imaging procedures within a 24 h window (median: 21 h; interquartile range 9–24 h). The demographics and ECMO characteristics of 17 patients are depicted in Table 1.

**Figure 1:**
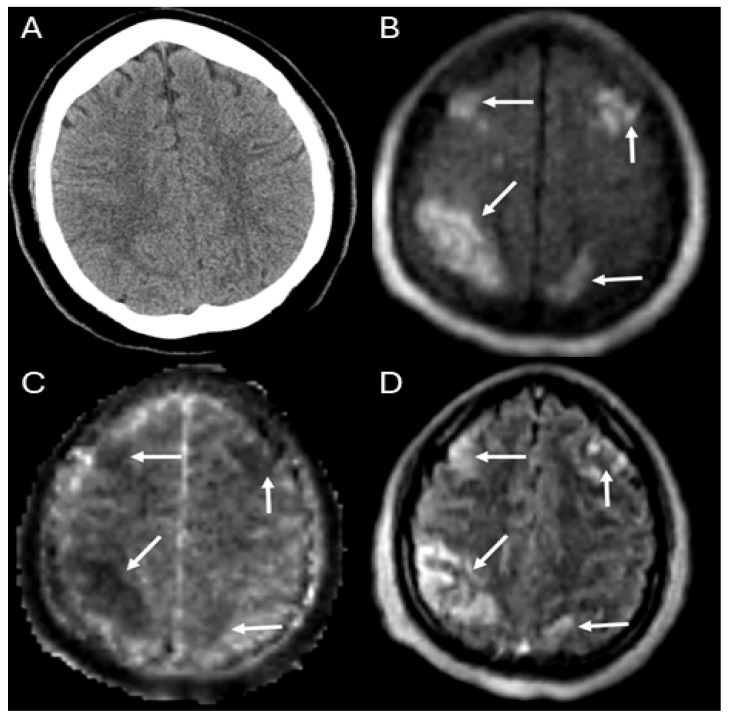
Example case: 31-year-old recently postpartum female with thrombotic thrombocytopenic purpura requiring extracorporeal cardiopulmonary resuscitation after cardiac arrest whose ULF-pMR demonstrated multifocal ischemic stroke (arrows: bilateral frontal and parietal strokes) on the Panels (**B**) (DWI), (**C**) (ADC), and (**D**) (FLAIR), not seen on the HCT (Panel (**A**)).

**Figure 2:**
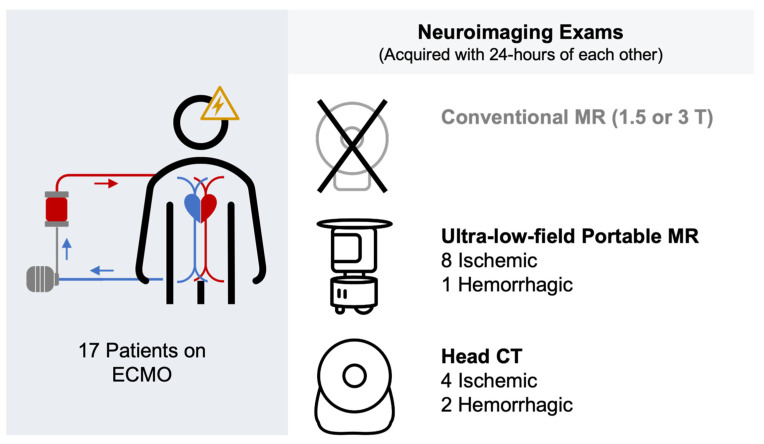
Detection of acute brain injury (ABI) events in patients on extracorporeal membrane oxygenation (ECMO) (Blue: drain cannula; Red: Return cannula) with MR and CT. Patients on ECMO cannot be safely scanned at conventional MR field strengths (1.5 or 3 T); therefore, ultra-low-field portable MR (ULF-pMR) and head CT (HCT) were acquired within 24 h of each other in 17 patients on ECMO. More ischemic ABI events were observed on ULF-pMR compared to HCT (8 vs. 4, respectively). Two hemorrhagic events were observed with HCT compared to one on ULF-pMR.

Of the 17 patients assessed, ABI was not observed on either ULF-PMR or HCT in 8 patients (47%). The remaining 9 patients (53%) had confirmed ABI with a total of 10 events (8 ischemic, 2 hemorrhagic). Detailed information on these 7 patients with ABI is in Supplemental Table S1. Of the 10 ABI events, 4 ischemic events and 1 hemorrhagic event were observed on both ULF-PMR and HCT (40% and 10%, respectively), 4 ischemic events were observed on ULF-PMR but not HCT (40%), and 1 hemorrhagic event was observed on HCT but not on ULF-PMR. Figure 1 shows an example where an ischemic event was visible on ULF-pMR but not seen on the associated HCT. Results are summarized in Table 2 and in Figure 2.

Using HCT as the reference standard, ULF-pMR detected ischemic events with 100% sensitivity (69% specificity, 95% confidence interval (CI): [0.44–0.94]; PPV = 0.5, 95% CI: [0.15–0.84]; and NPV = 1.0) and hemorrhagic events with 50% sensitivity, 95% CI: [0–1] (100% specificity; PPV = 1.0; and NPV = 0.94, 95% CI: [0.83–1.0]). ULF-pMR demonstrated a weak (κ = 0.51) and moderate (κ = 0.63) correlation with HCT for ischemic and hemorrhagic events, respectively.

## 4. Discussion

HCT is the standard for assessing ABI in critically ill patients in the ICU, especially for ECMO patients; however, sensitivity for detecting ischemic ABI events is poor relative to conventional MR (1.5 or 3 T) [20] and potential adverse events from transport to the imaging suite put these patients at additional risk. Our retrospective study suggests that ULF-pMR may be a potential solution, allowing for the timely detection of ABI at the patient’s bedside.

The feasibility of ULF-pMR in the adult [8,9,10,11,12,13,15,16,17,18] and pediatric [14,21] intensive care setting has been demonstrated. Additionally, we previously reported that adult patients on VA- and VV-ECMO support [12] and patients on VA-ECMO with intra-aortic balloon pump support [22] can be safely imaged with ULF-pMR at the bedside in the ICU setting. Sabir et al. [21] demonstrated the feasibility of ULF-pMR in a neonate and three children on extracorporeal life support. In addition to feasibility, the effectiveness of detecting ischemic events in patients in the ICU has been demonstrated. Mazurek et al. [17] reported that ULF-pMR had 80.4% sensitivity for detecting intracerebral hemorrhage. Yuen et al. [18] reported the sensitivity of individual ULF-pMR sequences: 98% for T2-weighted, 100% for FLAIR, and 86% for DWI. However, both conventional MR (1.5 or 3 T) and non-contrast CT were used as the reference standard for those assessments. In this small study, we found the sensitivity of ULF-pMR to be 100% for ischemic events and 50% (95% CI: 0–1.0) for hemorrhagic events; however, these values should be interpreted with caution given that conventional MR (1.5 or 3 T) is the reference standard for detecting ischemic events and HCT was used here and that only two hemorrhagic events were observed, limiting the statistical power.

In this retrospective analysis of a prospective multicenter study (SAFE MRI study) with convenient sample as a proof of principle, we observed that ULF-pMR was able to detect more ABI events (9 of 10 events) than HCT (6 of 10 events) for a cohort of patients in the ICU on extracorporeal life support. There were four ischemic events that were only detected by ULF-pMR and not seen on HCT. These results are consistent with preliminary results reported by Kuoy et al. [13] who reported acute infarctions seen on ULF-pMR and not on CT in 3 of 23 cases performed in the ICU. Although we are not able to draw any concrete conclusions based on this study due to the small sample size, these findings may indicate that ULF-pMR is more sensitive in ischemic ABI. While one hemorrhagic event was only seen in HCT but missed in ULF-pMR in this study, previous reports have demonstrated that ULF-pMR is able to detect hemorrhagic events [11,17] with a sensitivity of 80.4% [17].

There are several limitations to this study, including the following. First, although the patient cohort was limited to patients who underwent both ULF-pMR and HCT exams within a 24 h window, there is a chance that the ABI event occurred in the time period between the two exams. A prospective comparison of ULF-pMR and HCT acquired within a shorter time window (e.g., 2 h) would provide a more definitive assessment of the relative sensitives of these modalities for detecting ABI. Second, although one ULF-pMR exam revealed a hemorrhagic ABI event and Mazurek et al. [17] reported that ULF-pMR can be valuable for the evaluation of hemorrhage, the current ULF-pMR technology does not include gradient echo or susceptibility-weighted imaging sequences, which are generally sensitive to small hemorrhages. The addition of a sequence sensitive to small bleeds would be beneficial for a comprehensive assessment of ABI in patients in the ICU. Until that is available, a larger study assessing the sensitivity and specificity of the existing system and sequences to bleeds of varying sizes and stages (i.e., hyperacute to chronic) and basic research to better understand the properties of normal tissue and bleeds (e.g., *T*_1_ and *T*_2_ relaxation times, apparent diffusion coefficients, and magnetic susceptibilities) at ULF would be beneficial. Third, both ULF-pMR and HCT could have missed small ischemic infarcts such as punctate infarcts that may be only detected in traditional MRI. Finally, due to the limited number of patients and the retrospective nature of the study, cautious interpretation and larger prospective studies are necessary.

## 5. Conclusions and Future Work

ULF-pMR demonstrated an advantage over HCT in the detection of ABI in adult ICU patients on ECMO support within a 24 h window. Its potentially increased sensitivity, especially for ischemic ABI, makes it a promising choice for clinicians looking for a comprehensive cerebral evaluation in patients too sick to safely move or with contraindications for conventional MR. This work represents a step toward improving patient outcomes by providing earlier detection of ABI in critically ill patients so that brain-saving interventions may be applied in a timely manner.

## Figures and Tables

**Table 1:** Demographics and ECMO characteristics of 17 patients.

	Total (*n* = 17)
Demographics	
Age, years (interquartile range)	55 (41–63)
Male	8 (47%)
Body Mass Index, kg/m^2^	31.6 (27.3–35.5)
Race	
White	6 (35%)
Black	6 (35%)
Hispanic	2 (12%)
Asian	2 (12%)
Others	1 (6%)
Past medical history	
Ischemic stroke	2 (12%)
Intracranial hemorrhage	0 (0%)
Hypertension	7 (41%)
Hyperlipidemia	6 (35%)
Diabetes mellitus	5 (29%)
Heart failure	4 (24%)
Chronic kidney disease	7 (41%)
Atrial fibrillation	3 (18%)
Pre-ECMO variables	
Inotropic or vasoactive support	15 (88%)
Cardiac arrest	5 (29%)
Glasgow Coma Scale	6 (4–12.5)
SOFA score (on MRI day)	13 (11–14)
ECMO Cannulation	
VA-ECMO	
Central cannulation	2 (12%)
Peripheral cannulation	10 (59%)
VV-ECMO	
Single lumen cannulation	4 (24%)
Double lumen cannulation	1 (6%)
Mortality	10 (59%)
ECMO duration (days)	7 (4–14)
VA-ECMO duration (days)	4.5 (2.8–8.5)
VV-ECMO duration (days)	28 (11–30)

ECMO: Extracorporeal membrane oxygenation; SOFA: Sequential Organ Failure Assessment; VA-ECMO: venoarterial-ECMO; VV-ECMO: venovenous-ECMO.

**Table 2:** Count of acute brain injury events observed by ULF-pMR and Head CT in 17 patients. Nine patients had 10 acute brain injury events and 8 patients had both negative ULF-pMR and Head CT scans.

		HCT	
	ABI Present?	Yes	No
ULF-pMR	Yes	5 ^†^	4 ^‡^
	No	1 *	8

^†^ 4 ischemic events and 1 hemorrhagic event; ^‡^ 4 ischemic events; * 1 hemorrhagic event. HCT: Head computed tomography; ABI: Acute brain injury; ULF-pMR: Ultra-low-field portable magnetic resonance.

## Data Availability

The data presented in this study are not available on request from the corresponding author. The data are not available due to privacy statement adherence as specified in prospective study from which the data presented here were retrospectively analyzed.

## Supplementary Materials

The following supporting information can be downloaded at: https://www.mdpi.com/article/10.3390/diagnostics14060606/s1, Table S1: Characteristics of the 9 patients with detectable acute brain injury and the respective findings of each imaging modality.
