# Supplementary material for: Detection of Acute Brain Injury in Intensive Care Unit Patients on ECMO Support Using Ultra-Low-Field Portable MRI: A Retrospective Analysis Compared to Head CT
Source: Diagnostics (Basel). 2024 Mar 13;14(6):606. doi: 10.3390/diagnostics14060606 (PMC10968816; doi:10.3390/diagnostics14060606)
Supplement: Supplementary file 1 [file diagnostics-14-00606-s001.zip › diagnostics-2898277-supplementary.pdf]

Supplemental Table S1. Characteristics of the 9 patients with detectable acute brain injury and the respective findings of each imaging modality.

Age	Gender	Race	BMI	Neurological Diagnosis	CT Read	ULF-pMR Read
58	F	White	35.46	Ischemic Stroke (on MRI) and IPH (on CT scan)	Multiple supratentorial brain parenchymal hemorrhage.	Possible right frontal lobe acute infarct diffusion image. Right parietal encephalomalacia from old infarct.
72	M	White	44.8	Ischemic stroke (on MRI)	Increased effacement of the cerebral sulci bilaterally.	T2 signal abnormality in occipital lobes and left temporal land parietal lobe with diffusion restriction.
52	F	White	43.7	Ischemic stroke (on MRI)	No acute or subacute intracranial abnormalities.	Possible acute to subacute right frontal lobe infarct.
58	F	White	27.86	Ischemic stroke (on MRI and CT)	Small infarct in the left cerebellar hemisphere.	Acute to subacute left lateral cerebellar infarct
54	F	Asian	18.12	SAH (on MRI and CT)	Small volume subarachnoid hemorrhage in the right superior frontal sulcus.	T2 FLAIR images show abnormal signal in the right superior frontal sulcus.
65	M	Black	34.86	Both HIBI and Brain death (on MRI and CT)	Loss of gray-white matter distinction in the cerebral and cerebellum, without effacement of the peripheral subarachnoid space. This is suggestive of early hypoxic ischemic insult.	Suspicious areas of cortical restricted diffusion in bilateral cerebral hemispheres.
31	F	Hispanic	20.89	Both HIBI and Brain death (on MRI and CT)	Complete loss of gray-white differentiation and effacement of the ventricles and basal cisterns compatible with extensive global ischemic brain injury, cerebral edema and transtentorial herniation.	Bilateral cerebral cortex and deep gray matter DWI hyperintensity (which for the most part has corresponding ADC isointensity), with accentuated gray-white matter differentiation on T2/FLAIR images, correlate with the finding discussed on recent CT head exam, concerning for hypoxic ischemic injury. Diffuse cerebral edema and effacement of basal cisterns redemonstrated.

61	M	White	36.51	Ischemic stroke (on MRI and CT)	Hypodensities with local mass effect in bilateral occipital lobes and left parietal lobe are suggestive of developing posterior circulation ischemic infarcts.	Evolving recent bilateral PCA territory infarcts and a small lacunar infarct in the left periventricular frontal lobe (lenticulostriate perforator territory).
31	F	Black	34.69	Ischemic stroke (on MRI)	No acute intracranial abnormality.	Multiple acute embolic versus watershed infarcts in the bilateral superior frontal lobes, parietal lobes, parieto-occipital junctions and anterior aspect of right caudate nucleus.

M: male, F: female, BMI: body mass index; IPH: intraparenchymal hemorrhage; SAH: subarachnoid hemorrhage
